# New species of *Foleyellides* (Nematoda: Onchocercidae: Waltonellinae), parasite of *Lithobates brownorum* (Amphibia: Ranidae) from South-eastern Mexico and genetic barcodes of the Mexican species of the genus

**DOI:** 10.1007/s11230-023-10108-1

**Published:** 2023-07-30

**Authors:** Yanet Velázquez-Urrieta, María Guadalupe Velarde-Aguilar, Alejandro Oceguera-Figueroa, Virginia León-Règagnon

**Affiliations:** 1 Posgrado en Ciencias Biológicas, Instituto de Biología, Universidad Nacional Autónoma de México, Avenida Universidad 3000, Ciudad Universitaria, Coyoacán, C.P. 04510 Mexico City, Mexico; 2 Laboratorio de Helmintología, Departamento de Zoología, Instituto de Biología, Universidad Nacional Autónoma de México, Circuito Zona Deportiva s/n, Ciudad Universitaria, Copilco, Coyoacán, CP 04510 Mexico City, Mexico; 3 Estación de Biología Chamela, Instituto de Biología, Universidad Nacional Autónoma de México, A. P. 21, San Patricio, CP 48980 Jalisco, Mexico

## Abstract

Specimens of *Foleyellides* were collected from the body cavity of frogs in different regions of Mexico; *Lithobates brownorum* from Yucatán, Quintana Roo and Campeche*; L. megapoda* from Jalisco and *Rhinella marina*, from Guerrero. *Foleyellides calakmulesis* **n. sp.** is described based on specimens found parasitizing *L. brownorum.* The new species is distinguished from the other members of the genus by the combination of the following male characters: four pairs of caudal papillae different in size and the presence of a preanal plaque. Partial DNA sequences of the mitochondrial Cytochrome Oxidase C, subunit I of the four known Mexican species of *Foleyellides* and two potentially new species collected in this study were generated and compared, validating the erection of the new species.

## Introduction

Ochoterena and Caballero (1932) described *Chandlerella striatus* Ochoterena and Caballero, 1932 parasitizing the body cavity of *Lithobates montezumae* from central Mexico. A few years later, Caballero (1935) reassigned *C. striatus* as the type species of the new genus *Foleyellides* Caballero, 1935, which some authors considered synonym of *Foleyella* Seurat, 1917 (Anderson & Bain, 1976; López-Neyra, 1956; Witenerg & Gerichter, 1994; Yamaguti, 1961), or *Waltonella* Schacher, 1974 (Bain & Prod’hon, 1974) (for a detailed review see Romero-Mayén & León-Règagnon, 2016).

Esslinger (1986) re-examined specimens of *Foleyellides striatus* (Ochoterena and Caballero, 1932) Caballero, 1935 and reinstated *Foleyellides* as a valid taxon, currently including 11 species: *Foleyellides americana* (Walton, 1929); *F. brachyoptera* (Wehr and Causey, 1939); *F. confusa* Schmidt and Kuntz, 1969;* F. dolichoptera* (Wehr and Causey, 1939); *F. duboisi* (Gedoelst, 1916); *F. flexicauda* (Schacher and Crans, 1973); *F. malayensis* (Petit and Yen, 1979); *F. mayenae* Romero-Mayén and León-Règagnon, 2016; *F. ranae* (Walton, 1929); *F. rhinellae* García-Prieto, Ruiz-Torres, Osorio-Sarabia and Merlo-Serna, 2014; and the type species, *F. striatus.* Eight of these species are distributed in North America (five in the United States of America and three in Mexico); two more in Asia, and one in Africa.

Mitochondrial DNA sequences, in particular partial COI sequences, known as barcodes, have shown to be useful to differentiate species of nematodes (Lima-Monteiro et al., 2018; Powers et al., 2018; Siddall et al., 2012), given the morphological conservatism in some groups and high phenotypic plasticity in others (Nadler, 2002; Powers et al., 2011). Nevertheless, the only barcodes representing *Foleyellides* available in GenBank belong to *F. mayenae*. The aim of this study is to describe a new species of *Foleyellides* from southeast Mexico based on morphological and molecular evidence, as well as to ameliorate the lack of molecular information for this group available in public repositories, since partial sequences of the mitochondrial COI gene were generated for the four known Mexican species and two potentially new species collected in this study.

## Materials and methods

### Specimens collection

Specimens of *Lithobates brownorum* were collected in Yucatán, Quintana Roo and Campeche, Mexico, during June and July 2016 (table 1). Specimens were collected under the scientific collection permits FAUT0056 issued to VLR and SGPA/DGVS/ 02798/16 to AOF by Secretaría del Medio Ambiente y Recursos Naturales (SEMARNAT). Amphibians were captured using dip nets and euthanized by an overdose of sodium pentobarbital, dissected and examined under stereomicroscope; worms were placed in saline solution (0.65%) for 4–8 min and examined in vivo for distinctive morphological traits. For morphological study, specimens were fixed in hot ethanol (96%) and preserved in ethanol (70%). For molecular analyses, worms were fixed and preserved in ethanol (100%). Nematodes previously collected and identified as *F. striatus* of *L. megapoda* from Jalisco (July 2012) and *F. rhinellae* of *Rhinella marina* from Guerrero (August 2010) were also processed for molecular analyses (table 1).

**Table 1:** Hosts, localities and GenBank accession numbers of *Foleyellides* spp. and outgroups included in our analysis

Species	Definitive host	Locality	GenBank Accession number (COI)
***Foleyellides calakmulensis* n. sp.**	*Lithobates brownorum*	Campeche, Mexico Quintana Roo, Mexico Yucatan, Mexico	OR2645-50, 65-70 OR2659-64 OR2651-58, 71
*Foleyellides mayenae*	*Lithobates psilonota* *Lithobates pustulosa*	Jalisco, Mexico Nayarit, Mexico	KC130675 - 79 KC130681 - 86
*Foleyellides rhinellae*	*Rhinella marina*	Guerrero, Mexico	OR268888-89
*Foleyellides striatus*	*Lithobates megapoda*	Jalisco, Mexico	MZ662824 - 26
*Foleyellides* sp. 1	*Lithobates brownorum*	Campeche, Mexico	OR264573-76
*Foleyellides* sp. 2	*Lithobates brownorum*	Quintana Roo, Mexico	OR264577
*Icosiella neglecta*	*Pelophylax ridibunda*	Ukraine	KP760189
*Neofoleyellides boerewors*	*Sclerophrys garmani* *Sclerophrys gutturalis*	South Africa	MN663133 MN663139
*Neofoleyellides martini*	*Leptopelis natalensis*	South Africa	MW774895
*Neofoleyellides steyni*	*Amietia delalandii*	South Africa	MW598467
*Ochoterenella* sp. 1	*Rhinella granulosa*	Venezuela	KP760198
*Ochoterenella* sp. 2	*Rhinella marina*	Venezuela	KP760199
*Ochoterenella* sp. 3	*Phyllomedusa bicolor*	French Guyana	KP760197
*Onchocerca volvulus*	*Homo sapiens*	Italy	AM749285
*Oswaldofilaria chabaudi*	*Tropidurus torquatus*	Brazil	KP760204
*Oswaldofilaria petersi*	*Crocodilurus amazonicus*	Brazil	KP760205

### Morphological analyses

Specimens were cleared with glycerine for 24 h and mounted between coverslips; measurements are given in millimetres (unless otherwise indicated), with minimum and maximum, mean and standard deviation in parentheses. Drawings were made using a camera lucida attached to a microscope. Helminth specimens were deposited in the Colección Nacional de Helmintos (CNHE), Instituto de Biología, Universidad Nacional Autónoma de México, Mexico City. Host specimens were desposited in the Colección Nacional de Anfibios y Reptiles (CNAR). For scanning electron microscopy (SEM), specimens were dehydrated through a graded ethanol series, critical point dried with K850 Critical Point Drier (Emitech, Ashford, England), sputter-coated with gold with Q150R Modular Coating System (Quo´Rum, Ashford, England), and examined with a Hitachi SU1510 SEM (Hitachi, Tokyo, Japan) at the Laboratorio Nacional de la Biodiversidad (LANABIO), Instituto de Biología, Universidad Nacional Autónoma de México.

### Molecular analyses

For molecular analyses, total DNA was extracted using the Jena Bioscience kit, following the protocol provided by the company (Jena Bioscience, Jena, Germany). Amplification and sequencing of partial DNA sequences of the mitochondrial Cytochrome Oxidase C subunit I (COI) locus were carried out using a cocktail of six primers: forward (C_NemF1t1: NemF1_t1+NemF2_t1+NemF3_t1), reverse (C_NemR1_t1: NemR1_t1+NemR2_t1 +NemR3_t1) (Prosser et al., 2013). Thermal cycling conditions for amplification reactions were 94 C for 1 min, five cycles at 94 C for 45 s, 45 C for 40 s, 72 C for 1 min, followed by 35 cycles at 94 C for 40 s, 51 C for 40 s, 72 C for 1 min and a final extension at 72 C for 5 min. Sequencing reactions were accomplished using an ABI 3730xl Genetic Analyzer (Thermo Fisher Scientific, Waltham, Massachusetts, USA) at the LANABIO.

Sequences were edited and assembled using the program Geneious 5.1.7 (Biomatters Ltd. Auckland, New Zealand). For phylogenetic analyses, sequences of *Foleyellides mayenae* and other species included in Waltonellinae were downloaded from GenBank (table 1), *Icosiella neglecta*, *Oswaldofilaria petersi, Oswaldofilaria chabaudi* and *Onchocerca volvulus* were used as outgroup (table 1). DNA sequences were aligned using MAFFT (Katoh & Standley, 2013), with the default parameters. Uncorrected P distances were obtained in MEGA-X (Kumar et al., 2018).

The phylogenetic analysis was performed through Bayesian inference (BI), using Markov Chain Monte Carlo (MCMC) in Mr. Bayes V 3.1.2 (Ronquist et al., 2012). The appropriate model of evolution (GTR+1+Γ) was determined with jModeltest 0.1.1 (Posada, 2008). The chains run for 1,500,000 generations, sampling trees every 1,000 generations; the first 25% of the sampled trees were discarded according to Tracer V 1.5 (htt://beast.bio.ed.ac.uk/ tracer); consensus topology and posterior probability values were calculated from the remaining 75% of the trees.

## Results

### Description

**Onchocercidae Leiper, 1911**

***Foleyellides*** **Caballero,** 1935

***Foleyellides calakmulensis*** **n. sp.**

**Fig. 1:**
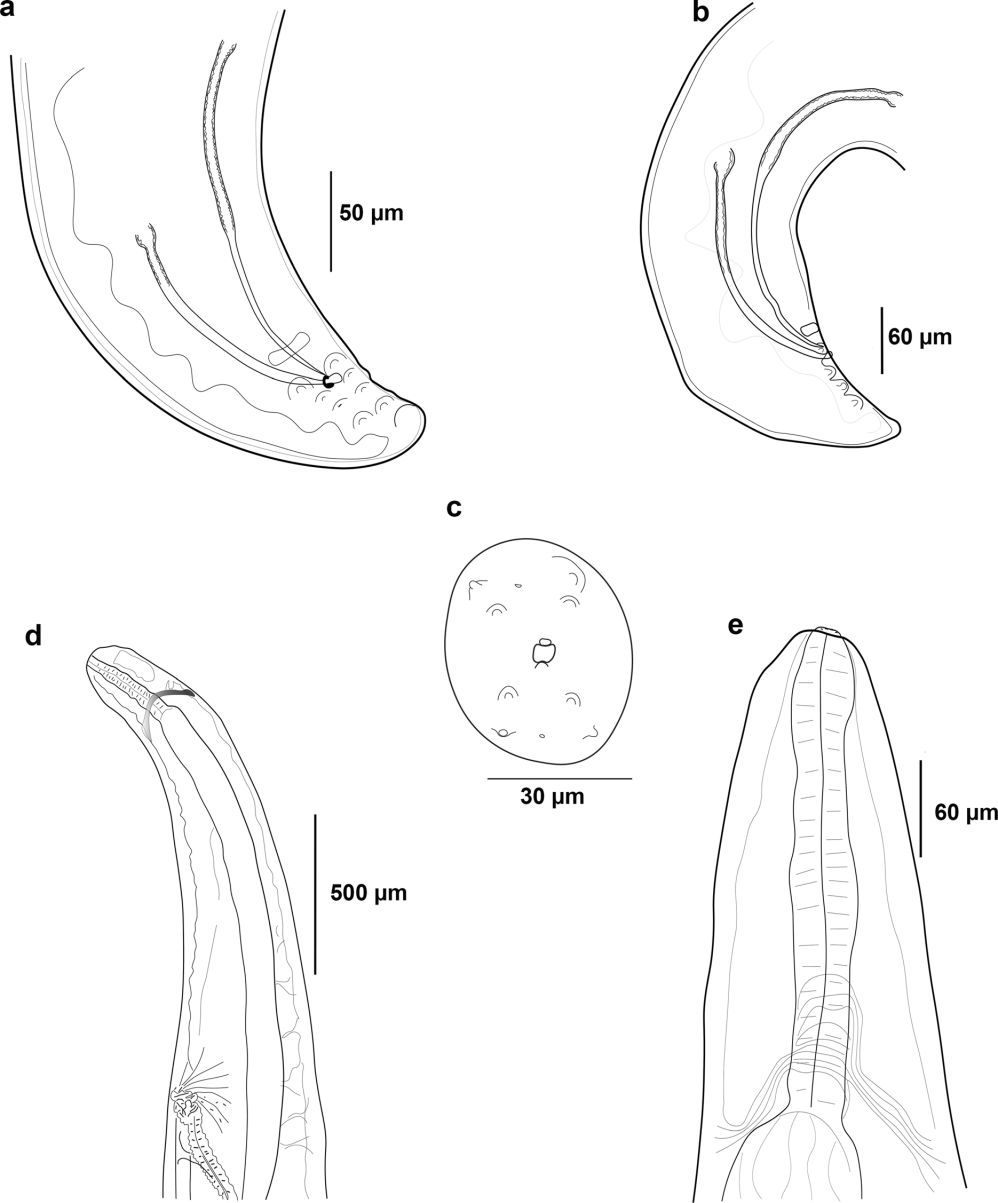
*Foleyellides calakmulensis* **n. sp.** Line drawing; male, ventral view of posterior end (a); male, lateral view of posterior end (b); apical view showing four pairs of papillae and parastomal structures (c); female, lateral view of anterior region showing position of vulva (d); male, ventral view of anterior end (e).

**Fig. 2:**
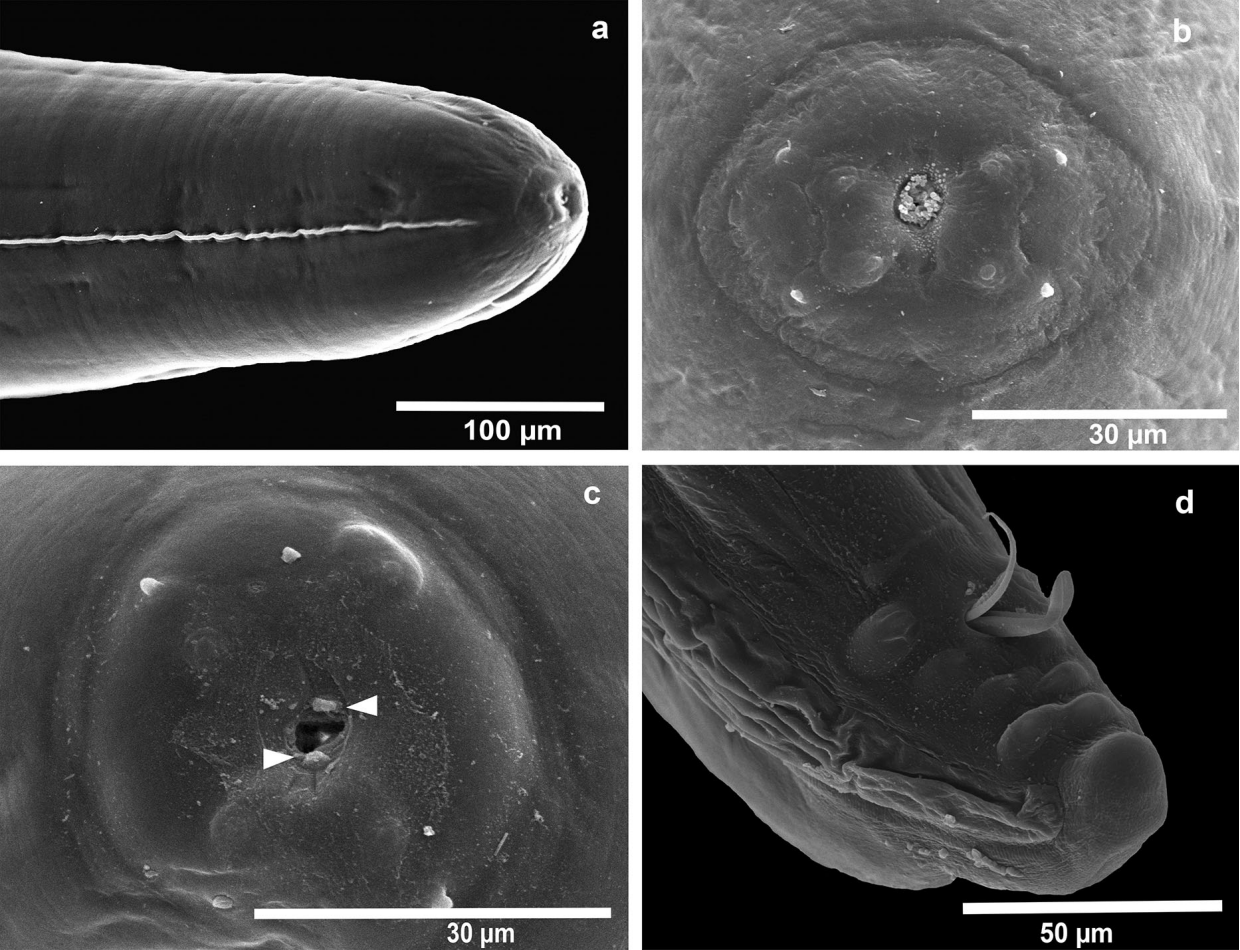
SEM of a male of *Foleyellides calakmulensis* **n. sp.** Lateral view of anterior region (a); apical view of anterior region showing four pairs of papillae (b); apical view showing parastomal structures (c); male, ventral view of caudal region, showing the distribution and size of papillae, preanal plaque and spicules (d).

**(**Fig. 1 & 2**)**

Male (*based on 18 mature specimens*): body length 12.57–20.26 (15.55 ± 1.84), wide at mid-hind body 0.21–0.40 (0.31±0.05). Maximum width at nerve ring level 0.15–0.20 (0.17 ± 0.016); at muscular/glandular esophagus junction 0.15–0.22 (0.18 ± 0.019) and at esophagus-intestinal junction 0.20–0.40 (0.30 ± 0.05). Cephalic extremity with one pair of cuticularized parastomal structures. Four pairs of cephalic papillae. Cephalic plate 30–60 (47 ± 6) µm long, 13–26 (20 ± 2) µm wide. Esophagus total length 1.301–2.12 (1.67 ± 0.22), muscular portion 0.17–0.40 (0.27 ± 0.06) long, 0.02–0.04 (0.03 ± 0.007) wide, glandular portion 1.11–1.80 (1.44 ± 1.16) long, 0.06–0.11 (0.08 ± 0.01) wide; ratio length glandular to muscular 1:0.123–0.256 (0.188 ± 0.03). Nerve ring 0.158–0.3 (0.22 ± 0.03) from anterior end. Tail length 0.08–0.17 (0.11 ± 0.03); dorsoventral thickness of body at level of anus 0.05–0.09 (0.07 ± 0.009). Four pairs of large and sessile caudal papillae; 1 pre-anal pair, 3 post-anal pairs; posterior pair 0.01–0.04 (0.02 ± 0.008) from tip of tail. Preanal cuticularized ventral plaque, well developed, thin and large, anterior to caudal papillae. Spicules unequal in form and size, right 125.3–247 (186.3 ± 32) µm long by 2.6–9 (4.9 ± 1.9) µm wide at base; left 265.3–375.3 (311.6 ± 32.2) µm long by 2.5–7.5 (5 ± 1.2) µm wide at base. Tail 0.08-0.17 (0.11 ± 0.03) long. Area rugosa as well as lateral and caudal alae well developed.

Female (*based on 12 gravid specimens*): body length 19.14–49.13 (31.57 ± 8.58); width at mid-hind body 0.27–0.77 (0.49 ± 0.15). Maximum width at nerve ring level 0.163–0.30 (0.13 ± 0.041); at muscular/glandular esophagus junction 0.17–0.31 (0.22 ± 0.04), at esophagus-intestinal junction 0.30–0.62 (0.44 ± 0.10). Cephalic extremity with one pair of cuticularized parastomal structures. Four pairs of cephalic papillae. Cephalic plate 38–65 (50 ± 8) µm long, 17–27 (21 ± 2) µm wide. Esophagus total length 1.39–2.73 (1.99 ± 0.36); muscular portion 0.22–0.31 (0.28 ± 0.02) long, 0.03–0.05 (0.04 ± 0.008) wide; glandular portion 1.14–2.41 (1.71 ± 0.34) long, 0.04–0.11 (0.08 ± 0.01) wide; ratio length glandular to muscular 1:0.12–0.22 (0.16 ± 0.025). Nerve ring 0.2–0.30 (0.23 ± 0.03) and vulva 1.23–1.95 (1.53 ± 0.20) from anterior end, respectively. Vagina uterine extending in the glandular region of esophagus, near to junction of muscular and glandular portions. Tail 0.36–0.68 (0.47 ± 0.11) long; width at anus level 0.36–1.14 (0.26 ± 0.07). Lateral and caudal alae present.

### Taxonomic summary

*Type host*: *Lithobates brownorum* Sanders. Specimens deposited: CNAR 31540–31544.

*Site of infection*: Body cavity.

*Type locality*: Calakmul, Campeche, Mexico (18º 48′ 25.9″ N, 89º 44′ 25″ W).

Other *localities:* Yum Balam, Quintana Roo; Lagunas de Yalahau, Yucatan

*Prevalence of infection*: 22 of 40 examined (55%).

*Type specimens deposited*: CNHE 11690, holotype, host CNAR 31542; CNHE 11691 & 11692, paratypes.

*GenBank accession number*: OR264545-71(COI)

*Zoo Bank registration*: 231B4DA5-D797-48CB-828F-3C683A0528E9

*Etymology*: The name of the new species refers to the collection locality, nearby the ancient Mayan city of Calakmul, Campeche, Mexico.

### Remarks

*Foleyellides calakmulensis* **n. sp.** is included in the genus based on morphological characters referred by Esslinger (1986) and Gibbons (2010), such as the presence of cuticularized parastomal structures, lateral and caudal alae in both sexes, and the lack of distinct cuticularized buccal capsule or annular bands of longitudinally oriented bosses on the cuticle of midbody region.

The new species differs from some other species of *Foleyellides* (*F. americana*, *F. brachyoptera, F. confusa, F. flexicauda, F. malayensis, F. mayenae, F. ranae,* and *F. rhinellae*) in the number of male caudal papillae, four pairs in the new species and more than four in the other species (García-Prieto et al., 2014; Romero-Mayén & León-Règagnon, 2016; Schmidt & Kuntz, 1969; Wehr & Causey, 1939; Witenerg & Gerichter, 1944). The new species also differs from *F. brachyoptera, F. confusa* and *F. mayenae* in the presence of a distinctive cuticularized preanal plaque, which is absent in those species (García-Prieto et al., 2014; Romero-Mayén & León-Règagnon, 2016; Schmidt & Kuntz, 1969). On the other hand, the new species resembles *F*. *duboisi*, *F. dolichoptera* and *F*. *striatus,* by having males with four pairs of caudal papillae (Esslinger, 1986; Wehr & Causey, 1939; Witenerg & Gerichter, 1944). However, in *F. duboisi* and *F. dolichoptera* the preanal plaque is absent, contrasting with *F*. *calakmulensis*. The new species most closely resembles *F*. *striatus* in the number of papillae and in the presence of a preanal plaque, but they differ in several features: 1) the size of females, which are smaller in the new species (38–74 in *F*. *striatus vs* 19–49 in the new species); 2) the difference in the size of left spicule, which is longer in *F*. *striatus* (336–465 *vs* 265 – 375); 3) the size of papillae (post-anal papillae are the same size in *F*. *striatus* (Esslinger, 1986), while different in size in the new species). These combined characteristics distinguish *F*. *calakmulensis* **n. sp.** from *F*. *striatus* and from the other described species of the genus.

### Genetic distances and phylogenetic analyses

**Fig. 3:**
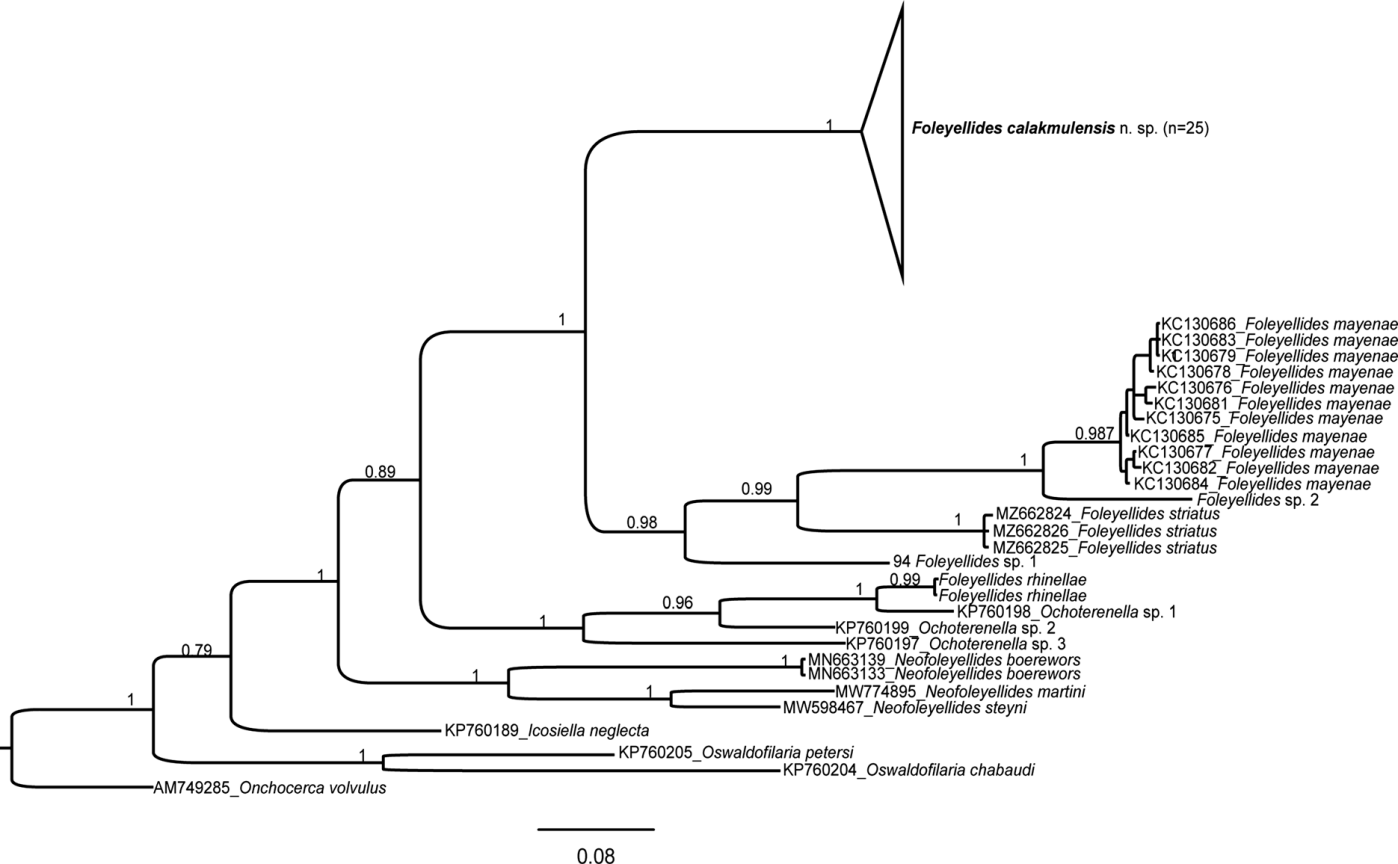
Bayesian phylogenetic tree of *Foleyellides* spp. based on COI sequences, showing the phylogenetic position of *Foleyellides calakmulensis* **n. sp.**; numbers above branches indicate posterior probabilities. The scale bar indicates the expected number of substitutions per site.

Specimens of two potentially new species of *Foleyellides* were collected during this study in Yucatan and Quintana Roo, Mexico (table 1); nevertheless, only female specimens were found in spite of intensive collecting efforts in both Mexican states. Without the male characters, those species can not be described in this study, and only COI sequences are presented. Genetic distances of the mitochondrial COI sequences of specimens of *F. calakmulensis* **n. sp.** from the same individual host and type locality range from 0 to 0.05%, and 0 to 0.70% between different localities. Genetic distances between *F. calakmulensis* and *F. striatus* range from 12.6–13.1%; 12.5–13.2 from *F. mayenae*, 15.5% from *F. rhinellae,* 10.5–11.5% from *Foleyellides* sp. 1 and 14.3–14.5% from *Foleyellides* sp. 2. Phylogenetic analysis results are presented in figure 3.

## Discussion

Eleven species of *Foleyellides* have been described in the world, the majority of which have been recorded in North America. Ten species are parasites of frogs of the family Ranidae and only *F. rhinellae* of toads (*Rhinella marina*) (García-Prieto et al., 2014); all of them inhabit the body cavity of the host, with exception of *F. confusa* which is subcutaneous (Schmidt & Kuntz, 1969). Taxonomy of the genus *Foleyellides* is mainly based on morphological characters (body size, number of caudal papillae, presence of cuticularized preanal plaque and size of the spicules); however, many of these characters are variable, and in some cases are difficult to distinguish between species. For example, males of *F. calakmulensis* **n. sp.** and *F. striatus* both have four pairs of papillae, and only with scanning electron microscopy it was possible to clearly corroborate that they are different in size (see Velarde-Aguilar, 2014).

In this sense, molecular tools are important for the differentiation and delimitation of species. We obtained COI sequences of *F. calakmulensis* **n. sp.**, *F. rhinellae, F. striatus, Foleyellides* sp. 1 and *Foleyellides* sp. 2 (table 1), and compared them with sequences of *F. mayenae,* which were the only available sequences of the genus in GenBank, in order to corroborate the validity of the new species. We also included sequences of other species in the subfamily Waltonellinae: *Neofoleyellides boerewors, N. steyni, N. martini* (Kuzmin et al., 2021; Netherlands et al., 2020) and three unidentified samples of *Ochoterenella* (Lefoulon et al., 2015). In the phylogenetic analysis, *F. calakmulensis* **n. sp.** appears within a highly supported clade that includes species collected from *Lithobates* spp. in Jalisco (*F. mayenae* and *F. striatus*), Yucatán (*Foleyellides* sp. 1) and Quintana Roo (*Foleyellides* sp. 2) (fig. 3). *Foleyellides rhinellae,* the only species of the genus described parasitizing toads, appears nested within samples of *Ochoterenella*, a genus that has been typically found in this group of hosts. Further morphological and molecular information would be needed to revise the phylogenetic position and taxonomy of *F. rhinellae* and to determine if this species should be transferred to *Ochoterenella*. All other species of *Foleyellides* included in the analysis are parasites of frogs in the genus *Lithobates.* It is interesting to note that species that share morphological traits (four pairs of papillae in *F. striatus* and *F. calakmulensis* **n. sp.**) or share geographical distribution and host species (*Foleyellides calakmulensis* **n. sp.**, *Foleyellides* sp. 1 and *Foleyellides* sp. 2, distributed in the Yucatan peninsula) are not sister species to each other in the tree. Further investigation on the phylogenetic relationships among species of Waltonaellinae is needed.

The new species is distributed only in south-eastern Mexico (Campeche, Quintana Roo and Yucatan), although additional geographic sampling is needed in order to determine the geographical distribution of the species in this genus, and also sequences of additional genes are needed to elucidate the evolution of morphological traits and host specificity of *Foleyellides* species.
